# Solid-State ^77^Se NMR of Organoselenium Compounds through Cross Polarization Magic Angle Spinning (CPMAS) Method

**DOI:** 10.1038/s41598-017-06892-8

**Published:** 2017-07-25

**Authors:** Duo Wei, Mengting Han, Lei Yu

**Affiliations:** 1 Testing Center, Yangzhou University, Yangzhou, 225008 China; 2 School of Chemistry and Chemical Engineering, Yangzhou University, Yangzhou, 225002 China

## Abstract

Characterization of selenium states by ^77^Se NMR is quite important to provide vital information for mechanism studies in organoselenium-catalyzed reactions. With the development of heterogeneous polymer-supported organoselenium catalysts, the solid state ^77^Se NMR comes to the spotlight. It is necessary to figure out an advanced protocol that provides good quality spectra within limited time because solid state ^77^Se NMR measurements are always time consuming due to the long relaxation time and the relatively low sensitivity. Studies on small molecules and several novel polymer-supported organoselenium materials in this article showed that cross polarization (CP) method with the assistance of magic angle spinning (MAS) was more efficient to get high quality spectra than the methods by using single pulse (SP) or high power ^1^H decoupling (HPHD) combined with MAS. These results lead to a good understanding of the effect of the molecular structure, the heteronuclear coupling, the long-range ordering of the solid (crystal or amorphous), and the symmetry of ^77^Se on quality of their spectra.

## Introduction

Selenium is a rare element first discovered by Swedish chemist Berzelius in 1818. It has been incorporated in nearly all areas of chemistry now because of the unique chemical and biological properties^1–6^. Recently, the catalytic activities of organoselenium compounds have attracted much attention^7–34^. Because selenium is a metabolizable element that will not accumulate in the body^35^, organoselenium catalysts are much safer for both organisms and the environment than transition metal catalysts. Organoselenium-catalyzed reactions usually employed hydrogen peroxide as the clean oxidant that generated no wastes other than the water^20–34^. Organoselenium catalysts were very stable and could be recycled and reused for many times without deactivation^24–34^. Binding onto polymers, the heterogeneous organoselenium catalysts were even easier to be separated from the reaction solutions and were of profound industrial application values^25^. During our continuous investigations on organoselenium catalysis^24–34^, we found that the selectivity of organoselenium-catalyzed reactions was tunable and different organoselenium catalysts might lead to different reaction paths^28^. Mechanism studies were very important to understand the protean reaction selectivity.

Besides control experiments,^77^Se NMR tests provided direct information of selenium states in reactions and were very efficient tools for mechanism study^24–27^. It has a wide chemical shift over 6000 ppm, which is beneficial for producing sufficient resolution to separate signals from different chemical sites^36, 37^. However, ^77^Se has a low natural abundance (*ca*. 7.63%) and low relative NMR sensitivity (6.93 × 10^−3^)^36–38^. The situation gets worse for solid-state NMR (SSNMR) especially when the Se content is even low and more complicated factors are involved. It is well known that the line width in SSNMR spectrum is usually wider than that in solution NMR because of the strong chemical shift anisotropy (CSA), dipolar-dipolar coupling, *etc*. Magic-angle spinning (MAS) is used routinely in the majority of SSNMR to remove the effects of CSA, to assist in the removal of heteronuclear dipolar-coupling effects, and to narrow lines from quadrupolar nuclei. Traditional single pulse (SP) sequence is always applied with the help of MAS, but still leads to low signal-noise ratio (SNR) for the characterization of ^77^Se.

The aim of the present article is to find out factors that affect the solid NMR spectrum and the best protocol to get high quality spectrum for several novel selenium-contained polymers. A method by using a combination of cross-polarization (CP) and MAS was applied, which has been previously used for the characterization of ^13^C, ^31^P etc^39–43^. The method offers a beneficial solution to achieve higher SNR based on the applications of polarization transfer from the abundant proton (^1^H) spin to low abundance nuclei i.e. ^77^Se in the present paper via heteronuclear dipole-dipole interactions. The improvements are originated from enhanced sensitivity of the Se and faster longitudinal relaxation time (*T*_*1*_) of ^1^H compared to ^77^Se leading to shorter recycle delay time (D_1_) between every acquisition (≥5*T*_*1*_)^44–46^. Experiments through traditional method of SP and a single pulse sequence with special high power ^1^H decoupling (HPHD) were also performed for comparison with the CP method. The CP method has been successfully applied to determine the state of the low-content selenium in several novel polymers. The novelty of the present paper is that the correlations of intensity of ^77^Se NMR signal with the solid state and molecular structure of polymers were demonstrated for the first time. Herein, we wish to report our findings.

## Results

**Figure 1:**
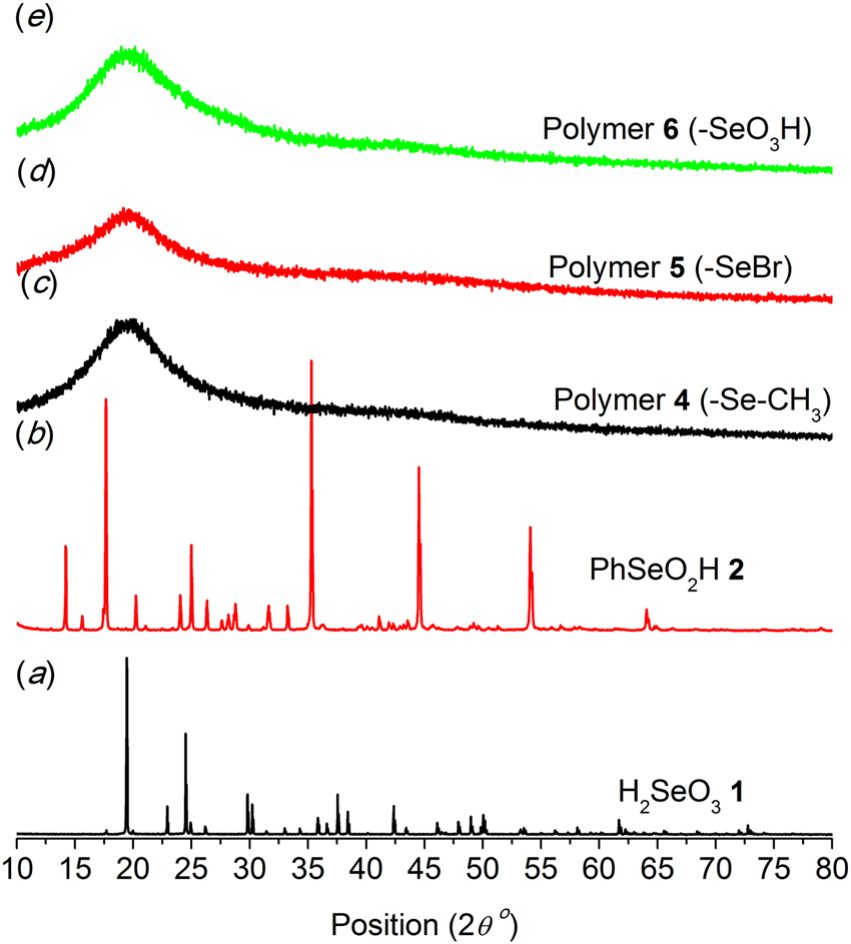
PXRD patterns: (**a**) H_2_SeO_3_ **1**; (**b**) PhSeO_2_H **2**; (**c–e**) Polymers **4**–**6**.

Solid-state properties of the samples were initially investigated. Figure 1 presents the powder X-ray diffraction (PXRD) patterns of the model compounds and polymer materials containing ^77^Se. PXRD pattern of H_2_SeO_3_ **1** is completely different from that of PhSeO_2_H **2**, confirming their different crystal structures due to the presence of a benzene ring^47^. In contrast, all the polymer materials exhibit a broad reflection, indicating their amorphous structures^48^.

**Figure 2:**
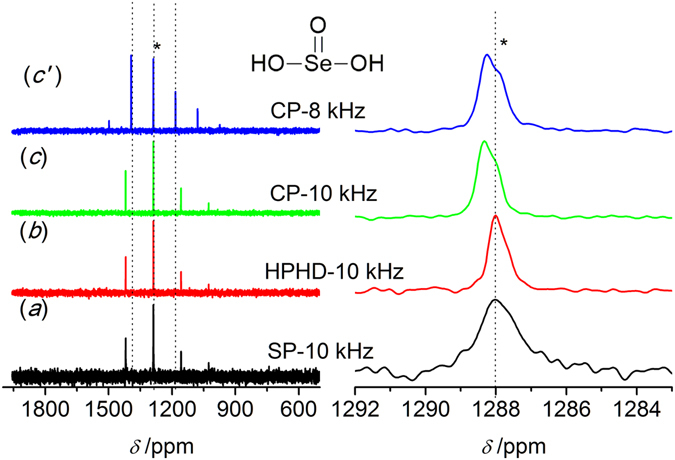
^77^Se MAS NMR spectra of H_2_SeO_3_ **1** obtained by different pulse sequences. (**a**) Single pulse (SP); (**b**) High power ^1^H decoupling (HPHD); (**c,** c′) Cross polarization (CP), 16 scans and the spinning speed of 10 kHz or 8 kHz. The isotropic peak is labeled with an asterisk (*).

To optimize the experimental parameters for different pulse sequences, H_2_SeO_3_ **1** was first measured and used as a secondary external reference standard. Figure 2 presents the ^77^Se NMR spectra obtained by the different pulse sequencesas described in experimental section: single pulse (SP), high power ^1^H decoupling (HPHD) and cross polarization (CP). To well illustrate the efficiency of the different methods, the intensity of the isotropic center-bands was normalized. A significant spinning side band manifold separated by the rotation frequency as a result of the chemical shift anisotropy (CSA) was observed in Fig. 2 left. The position of the isotropic center-bands (*) was determined by acquiring an additional spectrum at a different spinning speed of 8 kHz, which was enlarged in Fig. 2 right. The isotropic band remained at the same position for different speeds, while spinning side bands were changed (dashed lines, Fig. 2). Application of both HPHD and CP could enhance relative intensity of the resonance peak and narrow the lines (Fig. 2). An asymmetric isotropic band was observed for HPHD and CP, which might be related to defect in H_2_SeO_3_ crystal^49^. The chemical shift obtained by SP sequence was chosen to be the reference standard (1288.1 ppm). Full Width at Half Maxima (FWHM) and the SNR of the isotropic band were obtained and listed in Table 1. The SNR was improved from 13 for SP to 28 for HPHD and to 45 for CP, and the corresponding FWHM decreased from 101 Hz to 45 Hz and to 51 Hz. The existence of abundant ^1^H would broaden the line of ^77^Se spectra due to heteronuclear dipolar coupling between ^1^H and ^77^Se, which could be removed by ^1^H decoupling. Hence, the decoupling could improve SNR significantly and reduce FWHM, which explained the improvement for both HPHD and CP because of both of them including a decoupling pulse sequence. Moreover, the improvement for the CP was also related to the efficiency of polarization transfer between ^1^H and ^77^Se. It should be mentioned that CP needed less time to achieve comparable results as HPHD due to the shorter D_1_ (Table 1).

**Table 1:** Experimental parameters and chemical shift for different compounds.

Sample	Method	Number of scans	Delay time /s	Spinningspeed /kHz	SNR	FWHM
H_2_SeO_3_	SP	16	45	10	13	101
	HPHD	16	45	10	28	45
	CP	16	30	10	45	51
	CP	16	30	8	28	58
PhSeO_2_H	SP	1200	45	10	32	109
	HPHD	1200	45	10	45	82
	CP	1200	30	10	35	85
	CP	1200	20	8	17	79
Polymer **4** (-Se-CH_3_)	SP	17408	5	10	4	~1500
	HPHD	17408	5	10	5	~1800
	CP	17408	5	10	13	~1600
	CP	17108	5	13	16	~1800
Polymer **5** (-SeBr)	CP	17408	5	10	–	–
Polymer **6** (-SeO_3_H)	SP	17408	5	10	11	681
	HPHD	17408	5	10	11	698
	CP	17408	5	10	21	751
	CP	17408	5	13	21	742

**Figure 3:**
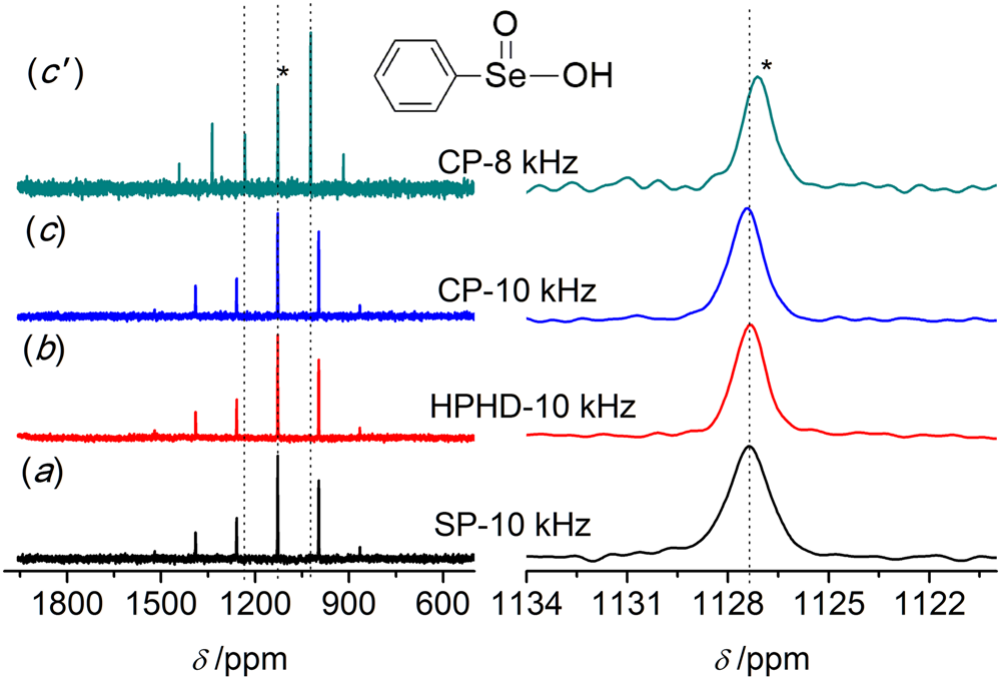
^77^Se MAS NMR spectra of PhSeO_2_H **2** obtained by different pulse sequences. (**a**) Single pulse (SP); (**b**) High power ^1^H decoupling (HPHD); (**c, c**′) Cross polarization (CP), 1200 scans and the spinning speed of 8 kHz or 10 kHz. The isotropic peak is labeled with an asterisk (*).

A pureorganic compound, PhSeO_2_H **2**, was used to justify the efficiency of the three pulse sequences, and the spectra of **2** obtained were displayed in Fig. 3. As shown in Fig. 3 left, more spinning side bands were observed compared to H_2_SeO_3_ **1**. The isotropic center-band was determined as marked by the asterisk (*), and its chemical shift was 1127.4 ppm with respect to H_2_SeO_3_ (Fig. 3 right). Similar to H_2_SeO_3_, HPHD and CP were able to enhance the relative intensity of the signal and narrow the line. The SNR was improved from 32 (SP) to 45 (HPHD) and to 35 (CP), and the FWHM was reduced from 109 Hz (SP) to 82 Hz (HPHD) and to 85 Hz (CP, Table 1). It was interesting that there was less enhancement of CP than that for H_2_SeO_3_. For H_2_SeO_3_, relatively strong hydrogen bond interactions induced the formation of well-ordered crystal and shortened the distance between Se atom and proton, whereas the presence of benzene ring in **2** distorted the hydrogen bond leading to total different crystal structure (Fig. 1) and consequently enlarged the distance between Se and proton^50^. These led to a weaker dipolar-dipolar interaction between ^77^Se and ^1^H spins for **2**, which explained the weaker enhancement of CP since the interaction was critical for cross polarization transfer efficiency. Moreover, low symmetry ^77^Se environments would have a large anisotropic shielding, i.e.large CSA, which led to a large number of spinning side bands^51^. Thus, the more spinning side bands for **2** might be attributed to lower symmetry ^77^Se environments compared to **1** as indicated from their molecular structures (Figs. 2 and 3).

**Figure 4:**
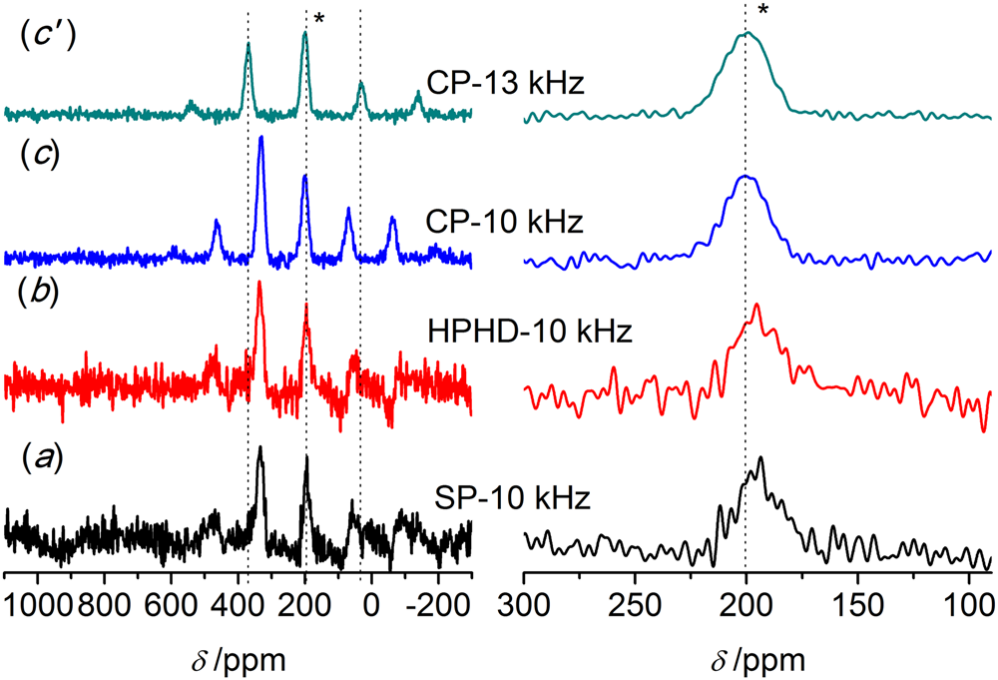
^77^Se MAS NMR spectra of polymer **4** (-Se-CH_3_) obtained by different pulse sequences. (**a**) Single pulse (SP); (**b**) High power ^1^H decoupling (HPHD); (**c, c**′) Cross polarization (CP), 17408 scans and the spinning speed of 10 kHz or 13 kHz. The isotropic peak is labeled with an asterisk (*).

The molar content of Se in polymer **6** (Scheme 1) was determined to be 1.46 mmol/g by an acid-base titration with NaOH. If all the benzenes were selenized, the Se content should be 4.33 mmol/g. Hence, only around 1/3 of benzenes in the polymer were substituted (Scheme 1). Accordingly, similar low-content of Se could be found in polymer **4** (-Se-CH_3_), which lead to low sensitivity of ^77^Se in NMR measurement. Figure 4 presents the spectra of polymer **4** obtained by the three methods. Several strong and wide spinning side bands could be observed in Fig. 4 left. The isotropic band (*) was determined by a faster speed acquisition (13 kHz), and its chemical shift was around 198 ppm (Fig. 4 right), close to values determined by solution NMR^36, 37^. In contrast to compounds **1** and **2** (Figs 2 and 3), only CP was able to effectively improve the relative intensity of the signals with SNR changing from 4 to 13 (Fig. 4 and Table 1). No obvious change happened to the FWHM value for HPHD and CP compared to SP, which might indicate weaker heteronuclear coupling. Thus, the improvement by using CP was originated to shorter relaxation time of ^1^H than ^77^Se and the cross polarization transfer efficiency. As indicated from Table 1, the FWHM of the isotropic band became much broader than those of compounds **1** and **2**, *i.e*. around 1600 Hz. The broadening of the ^77^Se peak might be attributed to chemical shift distribution^52^. The chemical shift distribution might be originated from the change in solid-state from more ordered crystalline to amorphous structures^38, 47, 48^. This explanation can be well supported by PXRD results (Fig. 1).

**Figure 5:**
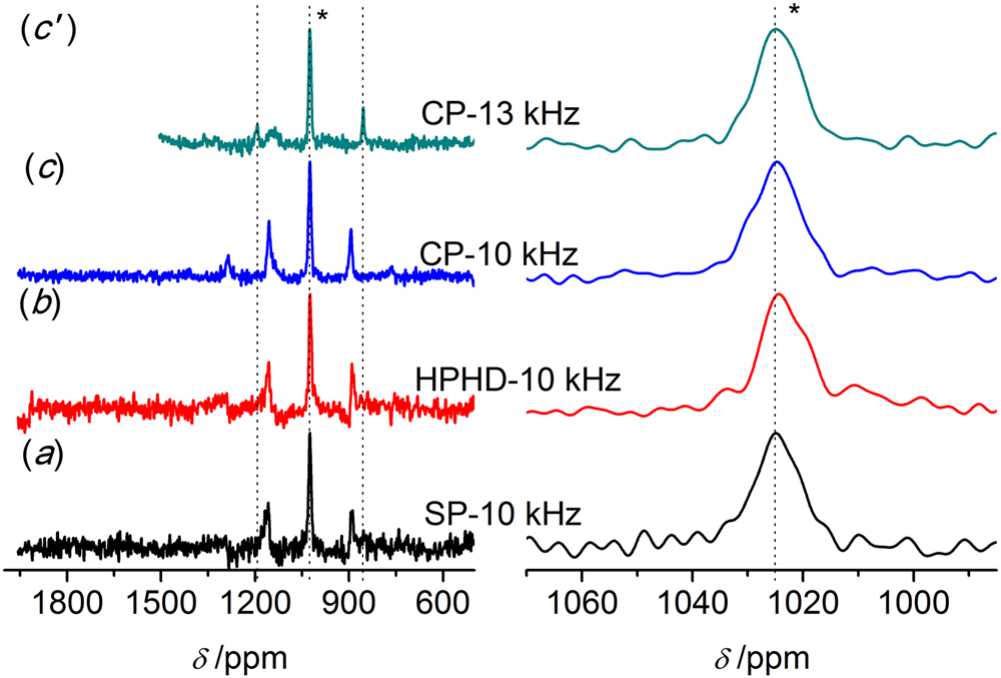
^77^Se MAS NMR spectra of polymer **6** (-SeO_3_H) obtained by different pulse sequences. (**a**) Single pulse(SP); (**b**) High power ^1^H decoupling (HPHD); (**c, c**′) Cross polarization (CP), 17408 scans and the spinning speed of 10 kHz or 13 kHz. The isotropic peak is labeled with an asterisk (*).

In order to determine the state of Se in different polymers, and gain effect of different substituted functional groups on the spectrum quality, polymer **5** (-SeBr) was measured by SSNMR with the same experimental parameters applied for polymer **4**. Both ^79^Br and ^81^Br are half-integer (I = 3/2) spins with large quadrupole moments (3.3 × 10^−25^ and 2.8 × 10^−25^ cm, respectively), which were so large that yieldedextensive line broadening^53^. As a result, the sensitivity of Se in this polymer was too weak to give a reliable result under the same conditions as polymer **4** (Data not shown). As for the polymer **6** (-SeO_3_H, Fig. 5), a well-defined single isotropic band and rather weak spinning side bands were observed. The isotropic band was determined to be 1023 ppm in line with reports (Fig. 5 left, *)^47^. Compared with polymer **4** (*ca*. 1600 Hz), the width of the isotropic bands became narrow (*ca*. 700 Hz, Table 1). The weak spinning side band might manifest higher symmetry ^77^Se environments in polymer **6** than that in polymer **4**, leading to lower CSA^51^. Thus, the relative intensity of the isotropic band was much stronger than polymer **4**showing higher SNReven for SP of 11 (Table 1). Although the polymers **4** and **6** were both in amorphous state (Fig. 1), the different line widths observed indicateddifferent motional properties and chemical shift distributions of Se atomas the substituted group changed from -Se-CH_3_ to -SeO_3_H^36, 37^. In addition, similar to polymer **4**, only CP could significantly enhance the relative intensity of signals (Figure 5left), and correspondingly improved the SNR values from 11 to 21 (Table 1).

**Figure 6:**
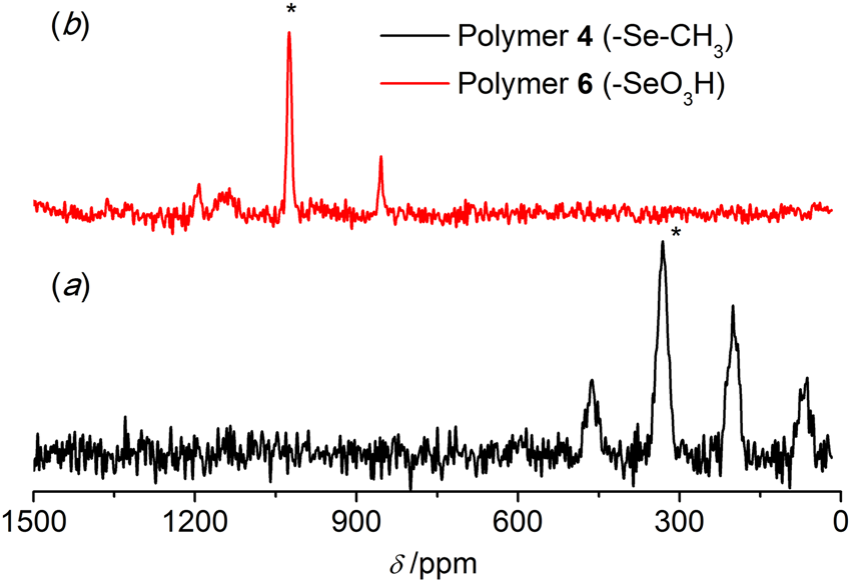
^77^Se CPMAS NMR spectra. (**a**) polymer **4** (-Se-CH_3_) and (**b**) polymer **6** (-SeO_3_H), 17408 scans and the spinning speed of 10 kHz or 13 kHz. The isotropic peak is labeled with an asterisk (*).

A comparison with the same spectrum range for polymer **4** and **6** was presented in Fig. 6. No detectable signal assigned to polymer **4** (*a*) could be observed in the spectrum of polymer **6** (*b*), indicating fully transformation from polymer **4** to polymer **6**, which manifested that SSNMR could characterize the Se state in polymers even under low content.

## Conclusion

The Se state in amorphous polymers even under low content was able to be efficiently determined by SSNMR through CP methods. It was found that low symmetry of molecular structure and less long range ordering structures of the solid would broaden the signal and enlarge the spinning side bands and conseqeuntly reduce SNR. These results would provide preliminary information for further investigation about the characterization of solidified selenium-containing materials from both molecular level perspective and macrostructure perspective.

## Methods

### Materials

**Figure 7:**
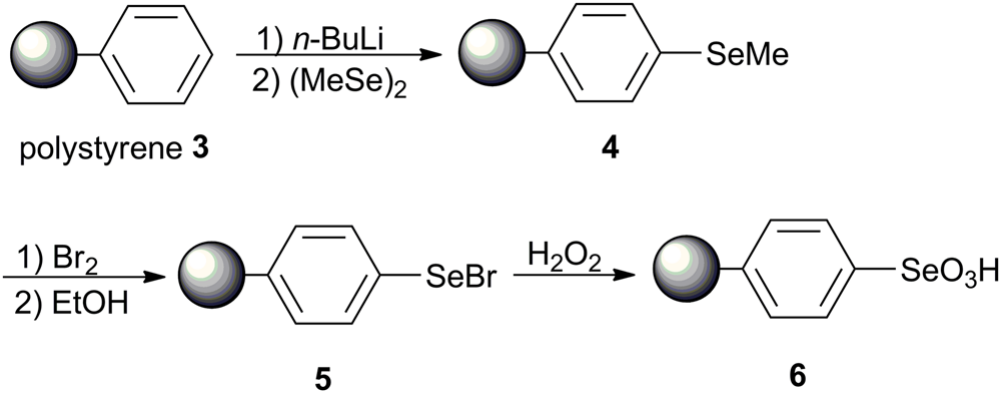
Preparation of the selenium-containing polymers **4–6**.

Seleninic acid **1** (H_2_SeO_3_) and benzeneseleninic acid **2** (PhSeO_2_H) were commercially available. The selenium-containing polymers **4**–**6** were prepared from polystyrene(Fig. 7).

### Detailed procedures for the preparation of polymer 4 from polystyrene 3

4.0 g of polystyrene resin **3** (1% cross-linked) was first immersed in cyclohexene (30 mL) overnight under N_2_. 5.6 mL of tetramethylethylenediamine (TMEDA, 37.2 mmol) and 48 mmol of *n*-BuLi (1.8 M, 26.7 mL) were added. The mixture was stirred at 65 °C for 4 h and filtrated. The polymer was then washed by 10 mL of THF for three times and removed into a flask by washing with 40 mL of THF and mixed with 5 mmol of (MeSe)_2_ at 0 °C. After stirring 20 min at 0 °C, water (3 mL) was added and the color of the polymer turned yellow. The polymer was washed with THF (15 mL, twice), H_2_O (15 mL, twice), methanol (15 mL, twice), CH_2_Cl_2_ (15 mL, twice) and ether (15 mL, twice) subsequently and dried under vacuum at 65 °C for 24 h to provide the polymer **4**.

### Detailed procedures for the preparation of polymer 5 from 4

4.0 g of polymer **4** was immersed in chloroform (60 mL) under N_2_ overnight. A solution of 6 mmol of Br_2_ in CHCl_3_ (20 mL) was added and stirred for 30 min cooled with ice-water.The resin was then washed with anhydrous EtOH and removed into a flask containing 60 mL of anhydrous EtOH. The color turned red after heating at 70 °C for 2 h and after filtration, the polymer was washed with EtOH (10 mL, twice) and CH_2_Cl_2_ (10 mL, twice) subsequently and then dried at 65 °C under vacuum for 24 h to produce the polymer **5** (4.3 g).

### Detailed procedures for the preparation of polymer 6 from 5

0.71 g of polymer **5** was immersed in 60 mL CHCl_3_ under N_2_ overnight. 10 mmol of H_2_O_2_ (30w/w %) was added at 0 °C. The color of the polymer turned white gradually after stirring at 25 °C for 2 h. After filtration, the resin was washed with EtOH (10 mL, twice) and CH_2_Cl_2_ (10 mL, twice) subsequently and then dried at 65 °C under vacuum to produce the pure polymer **6** (0.6849 g). Acid–base titration with NaOH indicated that the content of Se in polymer **6** was 1.46 mmol/g.

### Solid-state ^77^Se NMR

**Figure 8:**
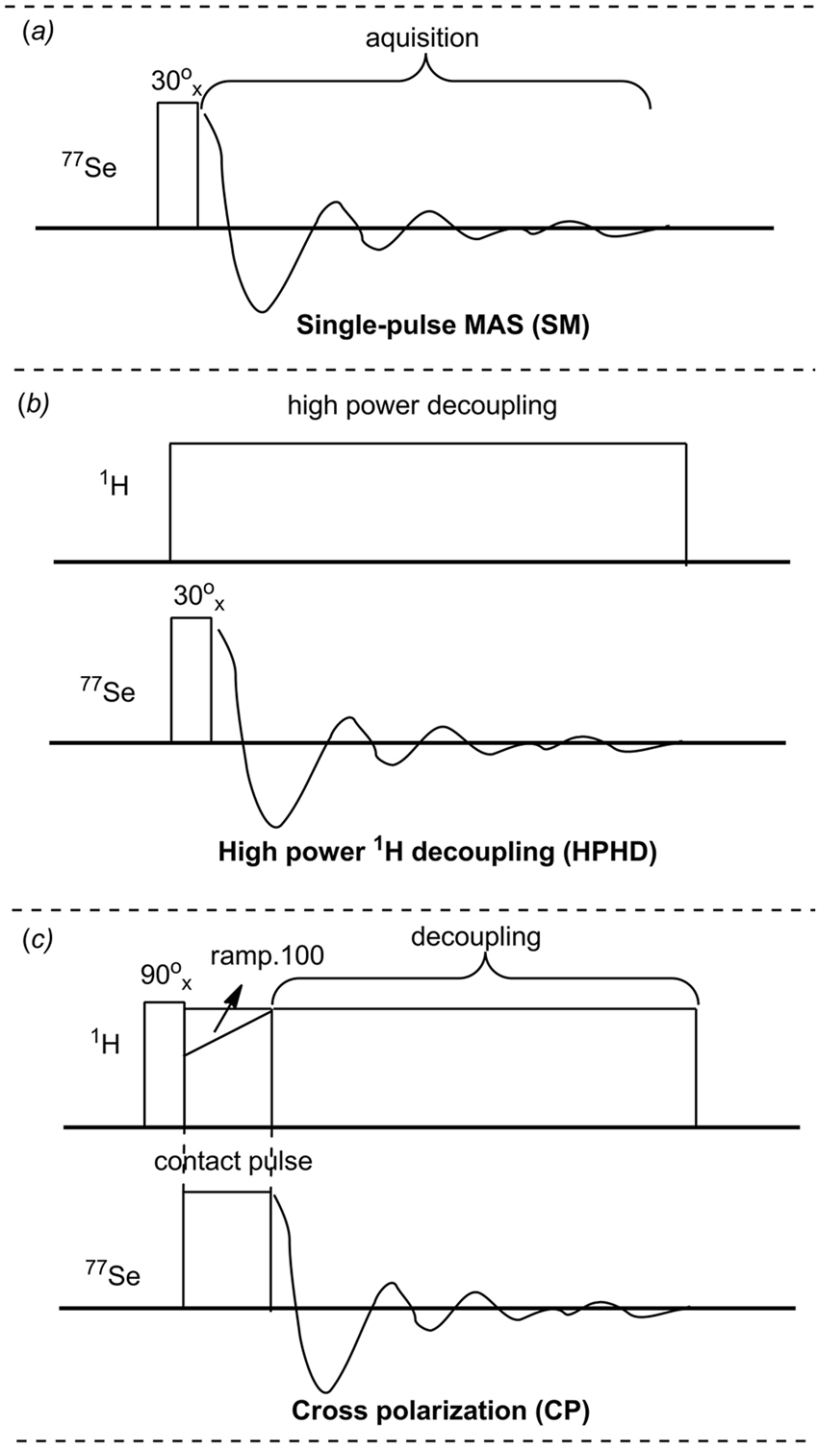
Schematic illustration of different pulse sequences. (**a**) Single pulse (SP); (**b**) High power ^1^H decoupling (HPHD); (**c**) Cross polarization (CP).

Solid-state ^77^Se NMR was performed on Bruker Avance III spectrometers operating magnetic field strengths of 9.4 T, corresponding to Larmor frequencies at 76.3 MHz for ^77^Se. A Bruker 4 mm double resonance HX MAS probe was used. H_2_SeO_3_ was used as a secondary external reference standard for PhSeO_2_H and polymers, and its isotropic chemical shift (*δ*) was calibrated to be 1288.1 ppm with respect to dimethylselenide (0.0 ppm). Three pulse sequences were applied (Fig. 8): 1, a single pulse (SP). 2, a single pulse with high power ^1^H decoupling (HPHD), and the composite pulse ^1^H decoupling program is spinal64.The decoupling power was set to be 66.72 W with the pulse length of 9.0 μs. 3, cross polarization (CP) transfer from ^1^H spin using optimized contact pulse durations of 4.5 ms or 5.0 ms (ramped for ^1^H), and two-pulse phase modulation (TPPM) ^1^H decoupling during acquisition (spinal64). The decoupling power was set to be 66.72 W with the pulse length of 8.4 μs. 90° radio frequency (rf) pulse length for^1^H excitation was 4.2 μs. In order to shorten recycle delay time (D_1_) between each acquisition, a 30°rf pulse of 1.54 μs (D_1_, *ca*. 0.1*T*_*1*_) was applied instead of the normally used 90° rf (D_1_, *ca*. 5*T*_*1*_) for ^77^Se excitation in SP and HPHD pulse sequences. The applied D_1_ are listed in Table 1. The acquisition time for all of the three pulse sequences was set to be 4.56 ms. Number of scans was varied from 16 to 17408 depending on the intensity of the samples. The data was acquired with spinning speed from 8 kHz to 13 kHz. The experiment durations of compound H_2_SeO_3_ and PhSeO_2_H are 12~15 min and 10~15 h, respectively, and the duration for the polymers is 24 h.
